# A tablet-based game for the assessment of visual motor skills in autistic children

**DOI:** 10.1038/s41746-023-00762-6

**Published:** 2023-02-03

**Authors:** Sam Perochon, J. Matias Di Martino, Kimberly L. H. Carpenter, Scott Compton, Naomi Davis, Steven Espinosa, Lauren Franz, Amber D. Rieder, Connor Sullivan, Guillermo Sapiro, Geraldine Dawson

**Affiliations:** 1 Department of Electrical and Computer Engineering, Duke University, Durham, NC USA; 2 Ecole Normale Supérieure Paris-Saclay, Gif-Sur-Yvette, France; 3 Department of Psychiatry and Behavioral Sciences, Duke University, Durham, NC USA; 4 Duke Center for Autism and Brain Development, Duke University, Durham, NC USA; 5 Office of Information Technology, Duke University, Durham, NC USA; 6 Duke Global Health Institute, Duke University, Durham, NC USA

**Keywords:** Diagnostic markers, Scientific data

## Abstract

Increasing evidence suggests that early motor impairments are a common feature of autism. Thus, scalable, quantitative methods for measuring motor behavior in young autistic children are needed. This work presents an engaging and scalable assessment of visual-motor abilities based on a bubble-popping game administered on a tablet. Participants are 233 children ranging from 1.5 to 10 years of age (147 neurotypical children and 86 children diagnosed with autism spectrum disorder [autistic], of which 32 are also diagnosed with co-occurring attention-deficit/hyperactivity disorder [autistic+ADHD]). Computer vision analyses are used to extract several game-based touch features, which are compared across autistic, autistic+ADHD, and neurotypical participants. Results show that younger (1.5-3 years) autistic children pop the bubbles at a lower rate, and their ability to touch the bubble’s center is less accurate compared to neurotypical children. When they pop a bubble, their finger lingers for a longer period, and they show more variability in their performance. In older children (3-10-years), consistent with previous research, the presence of co-occurring ADHD is associated with greater motor impairment, reflected in lower accuracy and more variable performance. Several motor features are correlated with standardized assessments of fine motor and cognitive abilities, as evaluated by an independent clinical assessment. These results highlight the potential of touch-based games as an efficient and scalable approach for assessing children’s visual-motor skills, which can be part of a broader screening tool for identifying early signs associated with autism.

## Introduction

Early detection of autism provides an opportunity for early intervention, which can improve developmental trajectories and strengthen social, language, cognitive, and motor competencies during a period of heightened brain plasticity^1–4^. The current standard of care for autism screening most often relies on a caregiver questionnaire, such as the Modified Checklist for Autism in Toddlers-Revised (MCHAT-R/F), which is used for neurodevelopmental screening in children between 16-30 months of age^5,6^. Although useful, the MCHAT-R/F has lower accuracy when administered in real-world settings, such as primary care^7,8^. Furthermore, the MCHAT-R/F’s performance is influenced by the family’s socioeconomic status, maternal education level, and the child’s sex, race, and ethnicity^7–10^. Thus, new objective screening and assessment tools based on direct assessment of the child’s behavior are needed that can complement screening approaches based on caregiver questionnaires.

While autism is fundamentally characterized by qualitative differences in social and communication domains, impairments in motor abilities have also been documented in autistic children^11–15^. The prevalence estimates of motor impairments in autism range from 50-85%;^14,16–19^ these estimates could potentially represent lower bounds since they are limited by the sensitivity of current assessment methods^15^. Motor impairments often are one of the earliest reported signs associated with autism^20–22^, and have been documented in autistic children without cognitive impairment^19^. Thus, early assessment of motor skills could be a useful component of an early screening battery for autism. Several aspects of motor skills have been studied in autism, including gait and balance stability, coordination, movement accuracy, reaction time, manual dexterity, tone, hyperkinesis, and praxis^15,21^. Various methods have been used to assess such skills using non-gamified paradigms, such as quantifying horizontal arm swings^23^, variations in reaching to grasp^24^ or touch^25^, handwriting^26^, and gait^27^.

Research suggests that differences in motor skills associated with autism emerge during infancy. LeBarton and Landa examined motor skills in 6-month-old infants with and without an older sibling with autism. Motor skills at 6 months predicted both an autism diagnosis and level of expressive language acquisition by 30-36 months^28^. These findings are consistent with other studies that have reported that the early development of motor skills is associated with expressive language outcomes among autistic children^29,30^. A recent study of patterns of health care utilization in infants who were later diagnosed with autism found a higher rate of physical therapy visits below age 1, underscoring the early manifestation of motor impairments in autism^31^.

Studies that have sought to characterize the nature of motor impairments in autism have found that autistic children are particularly challenged by tasks that require efficient visual-motor integration^32^. Visual-motor integration ability affects many domains of functioning, including imitation, which is fundamental for developing social skills. There is some evidence supporting a bias toward proprioceptive feedback over visual feedback in autism^33,34^. The tablet-based bubble-popping game developed for this study requires the temporal coordination of a dynamic visual stimulus with a motor response involving touch. As such, it is well suited to assess this aspect of early motor development.

The development of miniaturized inertial sensors, wearable sensors, and the ubiquity of mobile devices such as tablets and smartphones have allowed unprecedented access to massive multimodal data acquisition that has been used to characterize motor behavior. These data have been used to derive predictors of Parkinson’s severity^35^, identify and quantify an autism motor signature and characterize the nature of motor impairments in autism^36–45^. These studies demonstrate the usefulness of tablet-based assessments and games for assessing motor skills.

In the present study, we sought to extend current research findings in three ways. First, we sought to evaluate a tablet-based, gamified visual motor assessment in toddlers at the the age when autism screening is typically conducted. Second, intellectual abilities have been found to be correlated with motor impairment in autistic children;^24,46^ thus, we accounted for the contribution of co-occurring cognitive impairment to motor ability in our analyses. Third, as ADHD has also been associated with motor impairment, we sought to examine the combined contribution of autism and ADHD to the level and nature of motor impairment^47^. Previous studies have found that the prevalence of motor impairment among autistic individuals increases when there is co-occurring cognitive impairment and/or psychiatric conditions, including ADHD. One study found that the proportion of autistic children with motor impairment increased by 4.4% if the child had co-occurring ADHD. This study found that the nature of motor impairment in autism versus ADHD may differ, however^48^. Research suggests that, while autism has been associated with impairment in visual-proprioceptive integration, motor difficulties in ADHD tend to be associated with variability in the accuracy and speed of movement^34^.

The bubble popping game examined in this study is one part of a mobile application (app) developed by our team that displays developmentally appropriate and strategically designed movies while recording the child’s behavioral responses to the stimuli^49^. The app is administered on smartphones and tablets and does not require spoken language or literacy. Direct observation offers a unique opportunity for capturing and objectively quantifying various aspects of child behavior. We have previously reported results from children’s behavioral responses to the movies, which have been found to differentiate autistic from neurotypical toddlers^50–56^. In the current work, we focused on the bubble-popping game, which utilizes inertial and touch features. Based on previous studies, we predicted that autistic children would have a distinct performance on the bubble-popping game, and this pattern would differ between autistic children with versus without co-occurring ADHD. Additionally, we examined whether the motor digital phenotypes derived from the game correlated with standardized measures of cognitive, language, and motor abilities, as well as level of autism-related behaviors, to better understand the relationship between children’s motor behavior and their clinical profiles.

In summary, our goals were to: (i) assess motor behavior in children as young as 18 months using a tablet-based game to distinguish autism and neurotypical development at the age at which autism screening is typically conducted, (ii) control for the effects of cognitive ability in our analyses, (iii) evaluate the impact of co-occurring ADHD on motor function in young autistic children, and (iv) evaluate several novel visual-motor features derived from a simple, scalable game and their relationships with children’s clinical profiles.

## Results

### Correlations between motor performance and age

We first examined whether age of the participants was correlated with performance on the game. Combining samples from studies 1 and 2, results indicate that there was a strong correlation between the participant’s age and their game performance. Age has a significant positive association with the *number of touches* (rho = 0.62, *p* < 1e−25, *N* = 233) and the *bubble popping rate* (rho = 0.50, *p* < 1e−17, *N* = 233); and a significant negative association with the *median distance to the center* (rho = −0.48, *p* < 1e−16, *N* = 233), the *average touch duration* (rho = −0.70, *p* < 1e−36, *N* = 233) and the *average touch length* (rho = −0.63, *p* < 1e−28, *N* = 233). Given these associations, age was added as a covariate for all group comparisons and correlations in both studies.

### Study 1: Comparisons of younger autistic versus neurotypical children

**Fig. 1:**
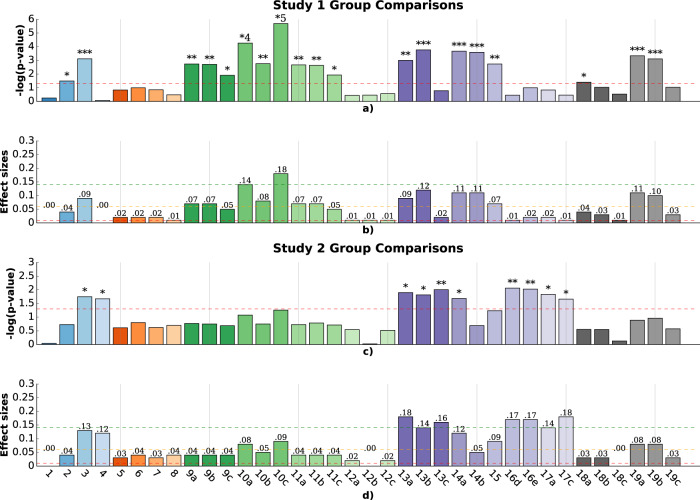
P-values and associated effect sizes for group comparisons of the touch-related features for autistic versus neurotypical participants in study 1, and autistic participants with and without co-occurring ADHD in study 2. (1) *number of touches*; (2) *number of pops*; (3) *bubble popping rate;* (4) *double touch rate*; (5) *screen exploratory percentage*; (6) *number of targeted*; (7) *number of transitions*; (8) *repeat percentage*; (9) *touch duration*; (10) *touch length of the touch motion*; (11) *touch velocity*; (12) *applied force*; (13) *distance to the center*; (14) *popping accuracy; (15) average variation of the popping accuracy; (16d) variability of the average popping accuracy*; *(16e) variability of the maximum popping accuracy*; (17) *number of touches per target*; (18) *touch frequency*; (19) *time spent on a targeted bubble*. **a** Mean; **b** Median; **c** Standard deviation; *p < 0.05; **p < 0.01; ***p < 0.001; *4:*p* < 0.00001; *5:*p* < 0.000001. *P*-values were computed using a one-way ANCOVA. Red dotted line indicates statistical significance at level 5%. *P*-values were corrected using Benjamini–Hochberg procedure to control for FDR. Red, orange, and green dotted lines indicate standard levels associated with a low (*η*^2^ = .01), middle (*η*^2^ = .04), and large (*η*^2^ = .14) effect size.

**Fig. 2:**
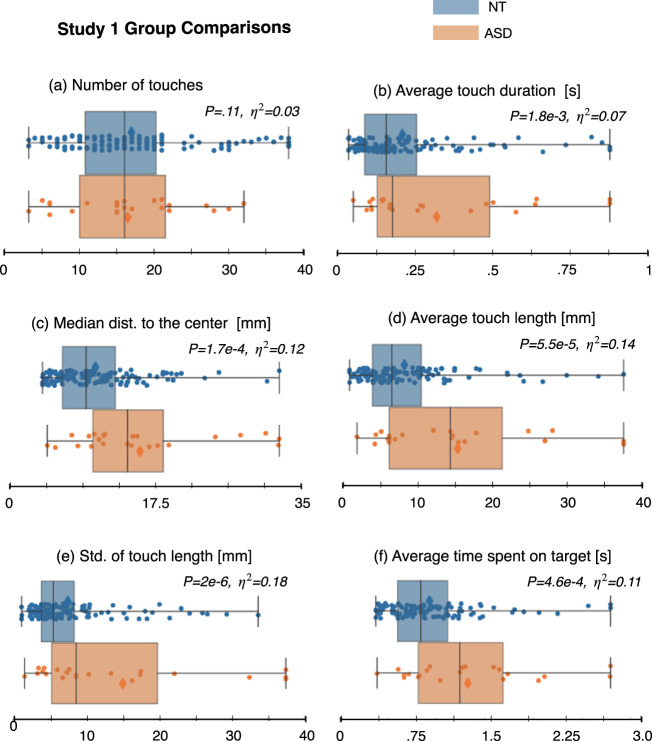
Group comparisons of distributions of several touch-related features for autistic versus neurotypical participants in study 1. These motor-related features show statistically significant differences between the groups (except for the *number of touches*). The extracted features presented here are detailed in the features extraction section. *P*-values were computed using a one-way ANCOVA, and corrected using Benjamini–Hochberg procedure to control for FDR. Effect sizes are denoted as *η*^2^. The line within the boxplot represents the median, the box represents the interquartile range, and the whiskers show extreme values. Scatter points show feature values for each participant.

Autistic and neurotypical participants in study 1 did not statistically differ in terms of their previous experience playing tablet-based games (Z = 0.96, *p* = .33; proportion Z-test). The level of engagement/compliance was not a significant factor, indicated by the high completion rate, higher than 95% for both groups. The age distribution comparison between the age-matched neurotypical group (*N* = 128) and autistic group was statistically non-significant (*p* = .07, *r* = .23; two-sided Mann–Whitney-U test). The two groups did not differ in terms of the mean number of touches, indicating similar levels of overall engagement with the game. However, the two groups were found to statistically differ in terms of several other motor variables. Figure 1 shows the p-values and effect sizes when comparing autistic and neurotypical toddlers on these touch-related features, and the data distribution of a subset of features are shown in Fig. 2 and Supplementary Fig. 1. Results showed that the autistic participants exhibited a lower *bubble popping rate* (Supplementary Fig. 1b, F(1, 148) = 15.14, *p* = 7.7e−4, *η*^2^ = 0.09), and their *median distance to the center* (mm) was larger (Fig. 2c, F(1,148) = 20.14, *p* = 1.7e−4, *η*^2^ = 0.12). Additionally, we observed that autistic participants had a longer *average touch length* (Fig. 2d, F(1, 148) = 23.56, *p* = 5.5e−5, *η*^2^ = 0.14), and showed greater variability in their touch length (Fig. 2e, F(1, 148) = 32,70, *p* = 2e−6, *η*^2^ = 0.18). We also found that the neurotypical participants took less time, on average, to pop a targeted bubble than autistic participants, as represented by the *average time spent to pop a bubble* (Fig. 2f, F(1, 148) = 18.56, *p* = 4.6e−4, *η*^2^ = 0.11). F-statistics and associated p-values were computed using a one-way ANCOVA.

### Study 2: Comparisons of older autistic versus neurotypical children

We first compared the autistic group (including those with co-occurring ADHD) and the neurotypical group in terms of their game performance. The two groups were found to differ in level of cognitive ability (*p* = 2e−5, *r* = 0.64; two-sided Mann–Whitney-U test) but not age (*p* = .15, *r* = .21; two-sided Mann–Whitney-U test); thus, we included both age and IQ, as reflected in their General Conceptual Ability (GCA) score, as covariates in these analyses. The level of engagement, as reflected in the mean number of touches, did not differ between autistic and neurotypical children (F (1,78) = .428, *p* = .77, *η*^2^ = 0.01; one-way ANCOVA). However, autistic children showed a significantly lower *average touch frequency* (F (1,57) = 14.77, *p* = 1.1e−2, *η*^2^ = 0.21), and a lower *median time spent targeting a bubble* (F (1,57) = 10.79, *p* = 2.0e−2, *η*^2^ = 0.16).

### Study 2: Comparisons of older autistic children with and without ADHD

**Fig. 3:**
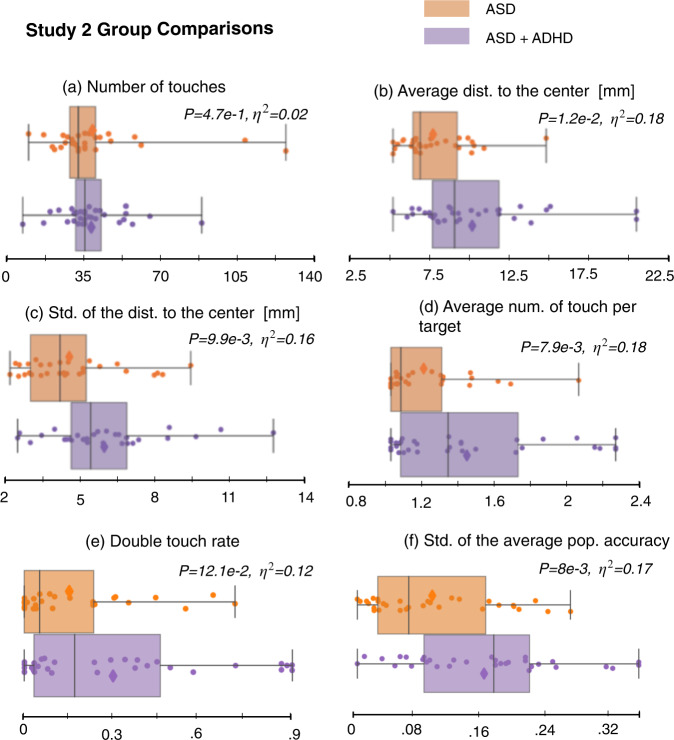
Group comparisons of distributions of several touch-related features for autistic versus autistic + ADHD participants in study 2. These motor-related features show statistically significant differences between the groups (except for the *number of touches*). The extracted features presented here are detailed in the features extraction section. *P*-values were computed using a one-way ANCOVA, and corrected using Benjamini–Hochberg procedure to control for FDR. Effect sizes are denoted as *η*^2^. The line within the boxplot represents the median, the box represents the interquartile range, and the whiskers show extreme values. Scatter points show feature values for each participant.

Children with and without ADHD did not differ in terms of age (*p* = .052, *r* = .28), previous experience playing video games (Z = −1.08, *p* = .28; proportion Z-test), or their cognitive ability (IQ) based on their GCA on the DAS (*p* = .68, *r* = .06; two-sided Mann–Whitney-U test). Figure 3 shows the distribution of a subset of touch-related features for the autistic participants with and without co-occurring ADHD. Fatigue/noncompliance was not a significant factor as the dropout rate for both groups was <.5%. Although the engagement in the task did not differ significantly between the autistic participants with and without ADHD, as indicated by the *number of touches* (Fig. 3a, F(1, 60) = 0.02, *p* = 0.90, *h*^2^ = 0.00; one-way ANCOVA), significant differences were observed in other motor features. Figure 1 shows the *p*-values and effect sizes when comparing children with and without ADHD on the touch-related features. Autistic participants with ADHD were, on average, less accurate as indicated by their *average distance to the center* (Fig. 3b, F(1, 60) = 12.76, *p* = 1.2e−2, *η*^2^ = 0.12), and consequently had a lower *bubble popping rate* (Supplementary Fig. 2a, F(1, 60) = 8.98, *p* = 1.7e−2, *η*^2^ = 0.13). Although the total *number of touches* did not differ, the group with ADHD showed higher *number of touches per target* (Fig. 3d, F (1, 60) = 10.0, p = 1.4e−2, *η*^2^ = 0.14). In addition, the group with ADHD showed more variability in their movement and accuracy. Specifically, they showed a higher *variability (std) in their number of touches per target* (Supplementary Fig. 2g, F(1,60) = 13.10, *p* = 2.1e−2, *η*^2^ = 0.18), *the distance to the center* (Fig. 3c, *F*(1,60) = 11.26, *p* = 9.9e−3, *η*^2^ = 0.16), and *the average popping accuracy* (Fig. 3f, F(1,60) = 12.71, *p* = 8.6e−3, *η*^2^ = 0.18). Additional results are presented in Supplementary Fig. 2.

### Combining features for group discrimination

**Fig. 4:**
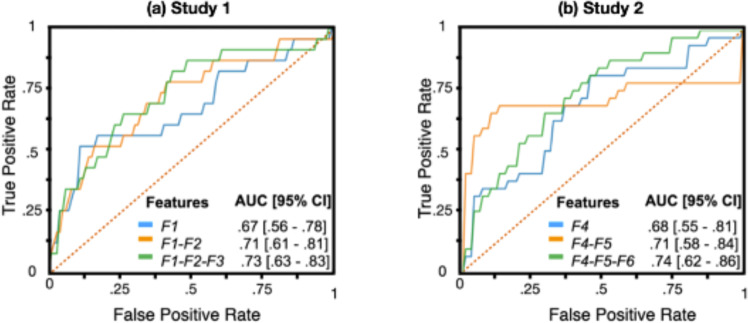
Receiver operating characteristic (ROC) curves and areas under the curve (AUC). ROCs and AUCs were obtained using logistic regression classifiers trained on a single, two, and three features, when differentiating autistic and neurotypical toddlers in study 1 (left) and autistic children aged 3-10 years with and without co-occurring ADHD in study 2 (right) samples. In both studies, level of group discrimination improves when adding features to the model. Confidence intervals were computed with the Hanley and McNeil method at 95% level. F1: *Average length* [mm]; F2: *Average touch duration* [s]; F3: *Average time spent* [s]; F4: *Average distance to the center* [mm]; F5: *Number of targets*]; F6: *Screen exploratory percentage*.

For study 1, we hypothesized that combining multiple features would improve discrimination of autistic and neurotypical toddlers. To this end, we trained logistic regression models to infer from the touch-based features the participant’s clinical diagnosis and performed leave-one-out cross-validation to assess the generalization performances of these models. We compared the performance of individual features and a combination of them to assess their complementariness. Figure 4 presents the receiver operating characteristic (ROC) curves and area under the curve (AUC) obtained for models trained by successively adding a single motor feature at a time. For study 1, the ROC shows the proportion of autistic participants correctly classified correctly vs. the proportion of autistic toddlers incorrectly classified by the model. Results showed that logistic regression trained on multiple game-based features improved the classification power; the AUCs using one, two, or three-motor features were 0.67 (95% CI, 0.56–0.78; *average length*), 0.71 (95% CI, 0.61–0.81; adding the *average touch duration*), and 0.73 (95% CI, 0.63–0.83; adding the *average time spent*), respectively.

For study 2, we also hypothesized that combining the motor-related features would improve group discrimination. The same previously described feature selection procedure was used. The ROC curve in Fig. 4b shows the proportion of Autistic+ADHD participants correctly classified vs. the proportion of autistic children incorrectly classified by the model. The AUCs using one, two or three motor features were 0.68 (95% CI, 0.55–0.81; *average distance to the center*), 0.74 (95% CI, 0.58–0.84; adding the *number of targets*), and 0.74 (95% CI, 0.62–0.86; adding the *screen exploratory percentage*), respectively. Supplementary Table 1 provides the AUCs obtained using three motor features by sex and racial/ethnic background. The AUC values remained relatively consistent for these subgroups; however, CIs were larger owing to smaller sample sizes.

### Study 1. Correlations between motor performance and clinical characteristics

**Fig. 5:**
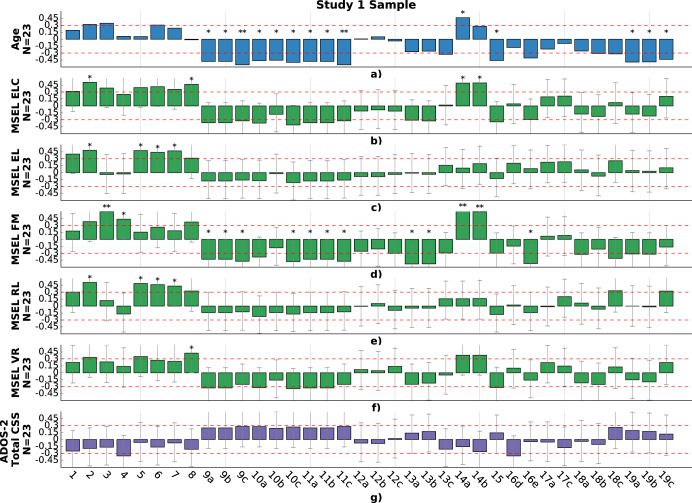
Correlations between computed motor-related variables (columns) and clinical measures (rows) for the study 1 sample. The height of the bar indicates the value of the partial correlation between a specific game variable and a clinical measure. (1) *number of touches*; (2) *number of pops*; (3) *bubble popping rate;* (4) *double touch rate*; (5) *screen exploratory percentage*; (6) *number of targeted*; (7) *number of transitions*; (8) *repeat percentage*; (9) *touch duration*; (10) *touch length of the touch motion*; (11) *touch velocity*; (12) *applied force*; (13) *distance to the center*; (14) *popping accuracy; (15) average variation of the popping accuracy; (16d) variability of the average popping accuracy*; *(16e) variability of the maximum popping accuracy*; (17) *number of touches per target*; (18) *touch frequency*; (19) *time spent on a targeted bubble*. **a** Mean; **b** Median; **c** Standard deviation; *p < 0.05; **p < 0.01; ***p < 0.001. *P*-values were computed using a Student’s t-test. Red dotted line indicates level of correlation of .3 and -.3. MSEL Mullen Scales of Early Learning, ELC Early Learning Composite Score, EL Expressive Language T-Score, FM Fine Motor T-Score, VR Visual Reception T-Score, ADOS-2 Autism Diagnostic Observation Schedule – Second Edition.

Spearman’s rho correlation was used to assess the relationship between motor features and clinical variables, with statistical significance computed using a Student’s t-distribution. We first examined the partial correlations between motor performance and the clinical characteristics based on clinician-administered measures, controlling for age, for the autistic children in study 1, including their performance on the Mullen Scales of Early Learning (MSEL) and the Autism Diagnostic Observation Schedule (ADOS total calibrated severity score). Partial correlations are illustrated in Fig. 5 for the autistic toddlers of the study 1 sample. The fine motor T-score of the MSEL was found to be positively correlated with the *pop rate* (rho = 0.59, *p* = 3.2e−3; Student’s t-distribution from now on), the *double touch rate* (rho = 0.43, *p* = 4.8e−2), and the *average popping accuracy* (rho = .62, *p* = 2.0e−3), and negatively correlated with the *average touch velocity* (rho = −.43, *p* = 4.5e−2), the *average* and *std touch duration* (rho = −.43, *p* = 4.5e−5 and rho = −.47, *p* = 2.5e−2 respectively), and the *variability of the maximum popping accuracy* (rho = −.52, *p* = 1.2e−2). The early learning composite score of the MSEL was found to be positively associated with the *number of pops* (rho = .51, *p* = 1.5e−2) and the *average popping accuracy* (rho = .49, *p* = 1.9e−2). The expressive language T-score of the MSEL was found to be positively correlated with the *screen exploratory percentage* (rho = .47, *p* = 2.5e−2) and the *total number of targets* (rho = .43, *p* = 2.1e−2). The receptive language T-score was positively associated with the *screen exploratory percentage* (rho = .48, *p* = 2.1e−2), and the visual reception T-score was positively correlated with the *repeat percentage* variable (rho = .42, *p* = 4.8e−2). No significant correlations were found between the motor features and the total calibrated severity score of the ADOS.

### Study 2. Correlations between motor performance and clinical characteristics

**Fig. 6:**
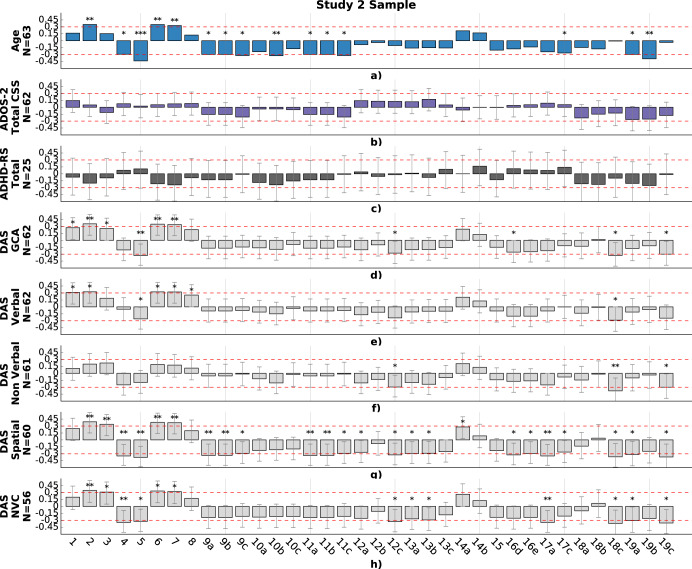
Correlations between computed motor-related variables (columns) and clinical measures (rows), for the study 2 sample. The height of the bar indicates the value of the partial correlation between a specific game variable and a clinical measure. (1) *number of touches*; (2) *number of pops*; (3) *bubble popping rate;* (4) *double touch rate*; (5) *screen exploratory percentage*; (6) *number of targeted*; (7) *number of transitions*; (8) *repeat percentage*; (9) *touch duration*; (10) *touch length of the touch motion*; (11) *touch velocity*; (12) *applied force*; (13) *distance to the center*; (14) *popping accuracy; (15) average variation of the popping accuracy; (16d) variability of the average popping accuracy*; *(16e) variability of the maximum popping accuracy*; (17) *number of touches per target*; (18) *touch frequency*; (19) *time spent on a targeted bubble*. **a** Mean; **b** Median; **c** Standard deviation; *P < 0.05; **P < 0.01; ***P < 0.001. Spearman’s rho correlation was used to assess the relationship between motor features and clinical variables, with statistical significance computed using a Student’s t-distribution. Red dotted line indicates level of correlation of .3 and −.3. ADOS-2 Autism Diagnostic Observation Schedule – Second Edition, ADHD-RS Attention Deficit/Hyperactivity Disorder Rating Scale, DAS Differential Abilities Scale, GCA General Conceptual Ability, NVC Non-Verbal Composite.

Spearman’s rho correlation was again used to assess the relationship between motor features and clinical variables, with statistical significance computed using a Student’s t-distribution. We examined the partial correlations between motor performance and the clinical characteristics, controlling for age, for the autistic children in study 2, including their performance on the ADOS total calibrated severity score, ADHD rating-scale total score, and the DAS. These analyses included children with and without co-occurring ADHD. Partial correlations are shown in Fig. 6 for the autistic children in the study 2 sample. We found that IQ was positively correlated with the *number of pops* (rho = .35, *p* = 4.9e–3; Student’s t-distribution from now on) and negatively correlated with the *screen exploratory percentage* (rho = −.34, *p* = 6e−3) and variability of the *touch frequency* (rho = −.32, *p* = .3e−2). The verbal standard score of the DAS was positivity correlated with the *number of touches* (rho = .31, *p* = 1.4e−2). The spatial standard score of the DAS was positively correlated with the *number of pops* (rho = .39, *p* = 2.1e−3) and negatively correlated with the *screen exploratory percentage* (rho = −.38, *p* = 3e−3), the *average touch duration* (rho = −.33, *p* = 9.1e−3), the *average touch velocity* (rho = −.33, *p* = 9.1e−3), the variation of the *force applied* (rho = −.32, *p* = 1.2e−2), and *the average time spent targeting a bubble* (rho = −.31, *p* = 3.1e−2). The non-verbal composite score of the DAS was positively correlated with the number of pops (rho = .34, *p* = 9.4e−3) and negatively correlated with the *double touches rate* (rho = −.34, *p* = 9.4e−3), the *screen exploratory percentage* (rho = −.32, *p* = 1.5e−2), and the *average time spent targeting a bubble* (rho = −.30, *p* = 4.5e−2). No significant correlations were found between the motor features and the total calibrated severity score of the ADOS.

## Discussion

Given increasing evidence of the role of motor impairments in autism, objective and accurate evaluation of fine motor skills is an important component of a comprehensive behavioral assessment of autism. We found that an easy-to-administer and engaging bubble popping game can collect meaningful, quantitative, and objective measures of early motor skills in children ranging from 18 months to 10 years of age. Data were feasibly collected in both clinical research settings and pediatric primary care clinics with minimal instructions, using a tablet and without special equipment and training. Therefore, this simple yet informative tool has the potential of being deployed at scale to enhance detection and assessment of early autism signs and obtain objective and quantitative measures of toddler and school age children’s visual motor skills.

Our results suggest that toddlers as young as 18 months old and children up to 10 years old showed a significant level of engagement with the game. Importantly, autistic and neurotypical children were equally likely to complete the game and touched the screen with similar frequency. In addition to a simple and engaging game that children of a wide age range can readily use, we engineered a set of touch and sensory-based features from the information recorded by the device. Features to evaluate the participants’ performance (e.g., number of touches, popping accuracy), their fine motor skills (e.g., popping accuracy, touch duration, applied force), and their preference for repetitive behaviors (e.g., repeat percentage, screen exploration) were measured.

We observed in both groups that several motor variables, including number of touches, bubble popping rate, median distance to the center, average touch duration, and average touch length, were correlated with age, suggesting that these features are promising as means to assess children’s developmental trajectories in visual motor skills. Even after controlling for age by matching groups on this variable and using age as a covariate, several differences in visual motor skills between autistic and neurotypical children emerged. In the younger toddler sample, autistic children popped the bubbles at a lower rate despite an equal number of touches, and their ability to touch the center of the bubble was less accurate. When they popped a bubble, their finger lingered for a longer period, consistent with previous findings^57^, and they showed more variability in their performance. In the older sample, compared to neurotypical children, the autistic children spent a longer period of time on a targeted bubble rather than moving quickly from one bubble to another. Consistent with previous research^47^, the presence of co-occurring ADHD was associated with lower visual motor skills. We found that autistic children with ADHD had lower accuracy (average distance from the center), lower number of pops despite an equal number of touches, higher number of touches per target, and overall, more variability in their motor behavior. These results are consistent with previous research showing that ADHD is associated with reduced visual motor accuracy and greater variability^34^. Finally, we proposed several game-based features and demonstrated that they can be aggregated in simple machine learning algorithms, trained to combine behavioral measurements to discover patterns that distinguish diagnostic groups, offering a potential to use such algorithms based on motor performance to differentiate toddlers and children with neurotypical development, autism, and those with or without co-occurring ADHD.

We also examined whether the motor features derived from the game showed meaningful correlations with independent clinical assessments of the autistic children. In autistic toddlers, several motor features were found to be correlated with the fine motor T-score on the Mullen scales, including pop rate and accuracy, double-touching, touch velocity and duration, and variability in touch popping accuracy (rho = −0.52). Overall IQ was found to be correlated with the number of pops and popping accuracy. Previous studies of infants who are later diagnosed with autism have found that early motor skills are associated with language acquisition^28–30^. We found that the number of different bubbles targeted during the game and the proportion of the screen explored by touch were positively associated with the expressive language T-score of the Mullen Scales. Interestingly, repetitive behavior during the game, reflected in the repeated popping of the same bubble, was positively associated with the Mullen visual reception T-score. It is possible that children with stronger visual perception skills were more likely to notice that the same bubble would appear after they popped it rather than quickly exploring other bubbles. Thus, the bubble-popping game might be able to identify visual perceptual strengths in autistic children. Finally, no associations between the motor features and level of autism-related behaviors on the ADOS were found in the toddler group.

In the older group, children with higher overall IQ, as well as those with higher spatial skills and nonverbal reasoning skills, tended to show stronger visual motor skills, as reflected in a greater number of bubbles popped as well as other features. Spatial skills measured on the Differential Abilities Scales, in particular, were consistently correlated with strong visual motor skills, as reflected in a higher number of bubbles popped, average touch duration and velocity, lower variation in the force applied, and average time spent targeting a bubble. Unlike in the younger sample of children, fewer correlations between motor features and language ability were found. Higher verbal skills were correlated only with the number of touches.

Gaming patterns hold promise for assessing children’s motor skills and potentially detecting early differences in motor behaviors associated with autism and ADHD. In the present study, we examined the distributions of the touch-based features and observed that many of the motor features differentiated autistic and neurotypical toddlers and autistic children with and without co-occurring ADHD. When comparing neurotypical and autistic participants, we observed that on average, neurotypical children exhibited greater visual motor control and accuracy. Both groups showed a similar level of engagement with the game (touching the screen a similar number of times). Still, neurotypical participants played the game with quicker and more accurate touches. Autistic children with co-occurring ADHD touched more of the screen and were less accurate and more variable in their motor responses. These findings underscore the role of co-occurring ADHD in accounting for variability in motor skills in autistic children.

Limitations of this work include the relatively limited number of participants to perform analysis per-demographic and per-sex groups. The relatively small sample size in autistic participants also limits the evaluation of the generalization ability of machine learning algorithms. Studies 1 and 2 had different clinical measures, limiting the possibility of comparing their relationship with motor variables on a broader sample. Longer games beyond 20 seconds might provide information about learning, focus, and anticipation. For study 1 of younger children, although it is possible that a child in the neurotypical group had an autism diagnosis, developmental or language delay, or both, it was not feasible to administer diagnostic and cognitive testing to all children. Children in the neurotypical group did not have a positive score on the M-CHAT-R/F and their parents and providers did not express a developmental concern.

This work and the informative data presented here are important steps towards characterizing the heterogeneity of motor functions in autism. Further work is needed to understand, differentiate, and disentangle motor differences associated with co-occurring psychiatric conditions. Additionally, leveraging ecological tools for the longitudinal quantification of motor function could be beneficial for the development of evidence-based interventions targeting visual motor impairments.

The tools proposed here are designed in the context of a broader effort to develop objective, digital behavioral phenotyping tools. Because children’s developmental trajectories are variable, it will be of interest to use digital phenotyping to longitudinally track a wider range of behaviors that can be captured with computer vision analysis, including gaze patterns/social attention^52^, facial expressions/dynamics^51,55^, postural control^58^, and fine motor control. The present study is a step in that direction. Future work includes evaluating the features proposed here in combination with others, advancing toward a multi-modal solution that objectively describes the rich and diverse realm of developmental variation precisely and quantitatively.

## Methods

### Participants

Study 1 was comprised of 151 children between 18 and 36 months of age, 23 of whom were subsequently diagnosed with autism spectrum disorder (ASD) based on DSM-5 criteria (see below). Children were recruited and assessed during their well-child visit at one of four Duke pediatric primary care clinics. Inclusion criteria were age of 16-38 months, not ill, and caregiver language was English or Spanish. Exclusion criteria were sensory or motor impairment that precluded sitting or viewing the app, parent not interested or did not have time to participate, child was too upset following doctor appointment, caregiver popped bubbles, or insufficient clinical information. From a larger group of neurotypical participants recruited for the study, neurotypical participants were selected randomly within the age range that matched the autistic group to limit any potential effects of age on analyses of group differences.

Study 2 was comprised of an independent sample of 82 children between 36 and 120 months of age. Based on a diagnostic evaluation (see below), of the 82 children, 63 had a DSM-5 diagnosis of ASD, of which 32 had co-occurring ADHD, and 19 were neurotypical (NT). Children were recruited from the community through flyers and brochures, emails, social media posts, and the research center’s registry. Inclusion criteria were aged 36-120 months, not ill, and caregiver language was English or Spanish. Exclusion criteria included a known genetic (e.g., fragile X) or neurological syndrome or condition with an established link to autism, history of epilepsy or seizure disorder (except for history of simple febrile seizures or if the child is seizure-free for the past year), motor or sensory impairment that would interfere with the valid completion of study measures, and history of neonatal brain damage (e.g., with diagnoses hypoxic or ischemic event).

In both studies, participants were excluded if the child did not understand the game (18 participants; NT = 13, Autistic = 5, Autistic+ADHD = 0; none of the study 2 participants failed to understand the game) or if caregivers popped the bubbles when the child was supposed to pop the bubbles by themselves (5 participants), as reported by the trained research assistant administering the app. Children who did not engage sufficiently in the game, defined as having touched the screen fewer than three times, were also excluded from the analysis (NT = 29, Autistic = 3, Autistic + ADHD = 0).

Table 1 describes the participants’ age, sex, and other demographic characteristics. Caregivers/legal guardians provided written informed consent, and the study was approved by the Duke University Health System Institutional Review Board (Pro00085434, Pro00085435, Pro00085156).

**Table 1:** Demographic characteristics of study sample.

Characteristics	*Study 1 (N* = *151)*	*Study 2 (N* = *82)*
Age (in months) - Mean (SD)	23.9 (3.20)	79.6 (15.65)
Sex – Total (%)		
Male	94 (62.5)	57 (69.5)
Female	57 (37.5)	25 (30.5)
Ethnicity – Total (%)		
Hispanic/Latino	16 (10.6)	9 (10.9)
Not Hispanic/Latino	135 (89.4)	73 (89.1)
Race -Total (%)		
American Indian/Alaskan Native	3 (1.9)	0 (0.0)
Asian	1 (0.6)	4 (4.8)
Black or African American	14 (9.3)	5 (6.0)
White/Caucasian	110 (72.8)	64 (78.0)
More than one race	17 (11.4)	7 (8.5)
Other	6 (4.0)	2 (2.7)
*Highest level of education - Total (%)*		
Without high school diploma	3 (2.0)	0 (0.0)
High school diploma or equivalent	8 (5.3)	2 (2.5)
Some college education	22 (14.6)	12 (14.6)
4-year college degree or more	118 (78.1)	68 (82.9)
*Familiarity playing game - Total (%)*		
Unknown/Not reported	1 (0.6)	13 (15.8)
Not at all	13 (8.6)	1 (1.2)
Rarely	89 (58.9)	11 (13.4)
Occasionally	22 (14.6)	15 (18.3)
Frequently	26 (17.3)	42 (51.3)
*ADOS calibrated total severity score*		
Unknown/not reported - Total (%)	128 (84.7)	20 (24.4)
Restricted and repetitive behavior CSS	8.39 (1.53)	9.1 (0.97)
Social affect CSS	7.17 (1.82)	7.31 (1.50)
Total CSS	7.78 (1.90)	8.11 (1.36)
Mullen Scales of Early Learning		
Unknown/Not reported - Total (%)	123 (81.4)	82 (100.0)
Early learning composite score	65.12 (11.79)	–
Expressive language T-score	29.30 (8.55)	–
Receptive language T-score	23.90 (5.61)	–
Fine motor T-score	34.00 (11.85)	–
Visual reception T-score	35.65 (11.99)	–
*ADHD-rating scale*		
Unknown/Not reported - Total (%)	151 (100.0)	47 (57.3)
Inattentive score	–	11.45 (8.10)
Hyperactive – impulsive score	–	11.77 (7.31)
Total score	–	23.22 (14.64)
Differential Abilities Scales		
Unknown/Not reported - total (%)	151 (100.0)	0 (0.0)
*General conceptual ability*	–	94.10 (23.89)
Verbal standard score	–	95.93 (25.78)
Non-verbal standard score	–	93.97 (18.31)
Spatial standard score	–	95.71 (22.63)
Special non-verbal composite standard score	–	94.02 (21.34)

*ADOS-2* Autism Diagnostic Observation Schedule – Second Edition, *CSS* Calibrated Severity Score.

### Clinical assessments

In study 1, at the time of app administration, caregivers also completed the Modified Checklist for Toddlers Revised with Follow-up (M-CHAT-R/F)^6^ during the well-child visit when the game was administered. M-CHAT-R/F is a caregiver-report screening questionnaire that asks about autism-related behaviors. Children who failed the M-CHAT-R/F and/or children for whom the caregiver or physician expressed a developmental concern were referred for a diagnostic evaluation conducted by a licensed and research-reliable psychologist. The average time between referral for evaluation and completing an evaluation was 3.5 months. The diagnostic evaluation included the Autism Diagnostic Observation Schedule - 2 (ADOS-2)^59^ and the Mullen Scales of Early Learning (MSEL)^60^, the latter of which yielded an Early Learning Composite Score (ELC) and the following subscale scores: (a) fine motor, (b) visual reception, (c) receptive language, and (d) expressive language. Children in study 1 were not evaluated for co-occurring ADHD because such a diagnosis is not considered reliable before age 3 years. Children were considered neurotypical if they did not fail the M-CHAT-R/F, and neither the caregiver nor their provider expressed a developmental concern. Neurotypical children did not receive a diagnostic or cognitive evaluation.

In study 2, an autism spectrum disorder diagnosis was established by a research-reliable clinical psychologist based on the ADOS-2 and the Autism Diagnostic Interview – Revised (ADI-R)^61^. Cognitive ability was assessed via the Differential Abilities Scale (DAS)^62^. Co-occurring DSM-5 ADHD diagnosis was established by a licensed clinical psychologist with expertise in ADHD (Davis) via the Mini-International Neuropsychiatric Interview for Children and Adolescents (MINI-Kid) with supplementary questions for assessing ADHD in children^63^, brief clinical child interview when appropriate, review of the parent-completed ADHD-Rating Scale (ADHD-RS)^64^, reviews of teacher-completed ADHD-RS when available, and clinical consensus based on clinical observations and these instruments. The ADHD-RS yielded an overall ADHD-RS score and Hyperactivity and Impulsivity subscale scores. For study 2, neurotypical children were defined as having an IQ > 70, Vineland Adaptive Behavior Scale scores in the average range^65^, and no clinical elevations on a set of parent-completed rating scales, including the Child Behavior Checklist^66^, ADHD-RS, and the Social Responsiveness Scale^67^.

Clinical data were collected using REDCap software.

### Pop the bubbles game

The bubble-popping game was delivered at the clinic directly following the well-child visit with the pediatrician. During the app, two types of stimuli are presented. First, a set of brief movies (in total, <10 min) with social and non-social content were displayed using the device’s screen. While the child watched the movies, the device’s frontal facing camera was used to capture their facial expressions, gaze, and postural/facial dynamics. Next, the bubble popping game was presented. Caregivers were asked to hold their child on their lap and the child was positioned such that they could independently and comfortably touch the iPad’s screen and play the game. The iPad was placed on a tripod, around 50 cm from the participant, allowing a sufficient dynamical response of the tripod when the touchscreen is touched while preserving the stability of the device. To minimize distractions during the app administration, other family members and the research staff were asked to stay behind both the caregiver and the child. First, the caregiver was encouraged to pop a few bubbles as a demonstration. Once the child had popped two bubbles independently, the training session ended, and the analyzed data began to be recorded for 20 seconds. By design, a bubble popped when the starting location of a touch was within 18.5 mm of its center. Furthermore, when the child popped a bubble, an identical bubble (i.e., same color) began to ascend from the bottom of the screen and came to the same location. This component of the game allowed an assessment of repetitive versus exploratory behavior (popping a different bubble than last popped). During the data collection, caregivers were instructed not to touch the screen nor provide any further instructions to the child. We used 7^th^ and 8^th^ generation iPads, both 10.2” inches. With a sampling rate of 60 Hz, on-device high precision inertial and gyroscopic sensors recorded the acceleration and orientation of the device, and screen-based features such as bubbles popping and screen touches. Inertial data were used to compute a proxy for the pressure applied on the screen. At the end of the game, caregivers were asked how frequently their child used tablets or smartphones; among those who responded (244/274, 89.1%), 94.3% of caregivers reported their child had previous experience watching or playing games on a tablet or smartphone (43% frequently, 33% occasionally, and 24% rarely).

### Feature extraction

**Fig. 7:**
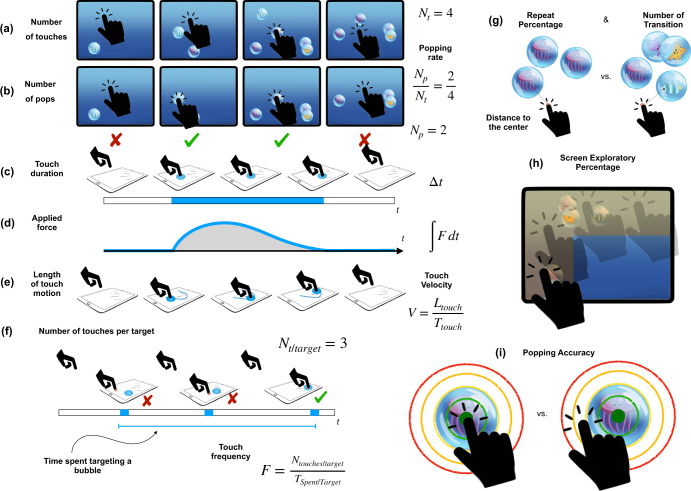
Illustration of the bubble popping game and the touch-based features extracted. This game is composed of 5 vertical tracks with bubbles appearing from the bottom and moving upwards. Any time a bubble is touched, the bubble pops, making a distinct popping sound releasing a cartoon animal character inside the bubble. When the bubble is popped, it appears again (same cartoon character) from the bottom of the same lane, otherwise a random one appears after the bubble exits the screen from the top. **a**–**i** graphically represent many of the touch-based features extracted from the game (see Methods).

Using the touch data collected and the tablet kinetic information provided by the device sensors, we computed a set of features representing the participants’ motor behavior. More precisely we defined: (1) *number of touches*, representing the total number of unique times the participant touched the screen, see Fig. 7a; (2) *number of pops*, the number of bubbles successfully popped, Fig. 7b; (3) *bubble popping rate*, the ratio of popped bubbles over the number of touches, Fig. 7b; (4) *double touch rate*, number of times the child tried to double touch the screen over the total number of touches; (5) *screen exploratory percentage*, proportion of area of the screen that was explored by the child’s touches, Fig. 7h; (6) *number of targeted bubbles*, representing the total number of bubbles that were targeted during the game, with a target defined as a bubble that is close enough to the location of a child’s touch; (7) *number of transitions*, number of times a different type of bubble (different lane) was popped; (8) *repeat percentage*, percentage of repeated bubbles (same lane and animal character) consecutively popped, Fig. 7g; (9) *average/median/std touch duration*, mean/median/standard deviation of the touches, that is, time the finger is on the screen during a touch, Fig. 7c; (10) *average/median/std touch length of the touch motion*, mean/median/standard deviation of the spatial length of the touches, Fig. 7e; (11) *average/median/std touch velocity*, mean/median/standard deviation of the ratio the *touch length* and the *touch duration*, Fig. 7e; (12) *average/median/std applied force*, approximated by computing the integral of the square of the acceleration of the iPad over the touch duration, retrieved from the built-in device accelerometers (see Fig. 7d and Supplementary Algorithms 1 and 2, and Supplementary Fig. 3 for additional details); (13) *average/median/std distance to the center*, mean/average/standard deviation of the distance between the finger impact location and the center of the popped bubble, Fig. 7g; (14) *average/median/std popping accuracy*, for a touch motion, a measure of spatial accuracy. Specifically, for each sample of a touch motion, we measured how far it was from the bubble area, with 100% accuracy defined as located on the bubble area and decreasing accuracy reflecting distances farther from the bubble edges. We then computed the mean/median/standard deviation of this measure across touches; (15) the *average variation of the popping accuracy* represents the mean standard deviation of the popping accuracy, across all touches, and the *variability of the average;* *(16d) popping accuracy represents* the standard deviation of the *average* *popping accuracy*, across all touches (maximum; 16e). See additional information on the popping accuracy in Fig. 7i and on Supplementary Fig. 4; (17) *number of touches per target*, representing the total number of time the participant hit near or on a bubble before it disappeared, Fig. 7f; (18) *average/median/std touch frequency (touch/s)*, representing the number of touches per second while targeting a bubble, Fig. 7f; (19) *average/median/std time spent on a targeted bubble*, mean/median/standard deviation of the time a targeted bubble was touched, Fig. 7f. See additional illustrations of the extracted features in Fig. 7 and Supplementary Fig. 5.

### Statistical analysis

Differences in previous experience with electronic games were assessed using a proportion Z-test. Group differences in age and IQ were assessed using a two-sided Mann–Whitney-U test. Effect size, denoted as’r’, was evaluated with the rank-biserial correlation algorithm^68^. Spearman’s rho correlation was used to assess the relationship between motor features and clinical variables, with statistical significance computed using a Student’s t-distribution^69^. Group comparisons were made using one-way ANCOVA for motor-related variables, with the diagnostic group as the categorical predictor (autistic/NT and autistic/ADHD + autistic). We used age as a covariate for study 1 sample, and age and IQ as covariates for study 2. Eta-squared, denoted as *η*^2^, was calculated to quantify effect sizes. Benjamini–Hochberg correction was applied to p-values to control for False Discovery Rate (FDR)^68^. Significance was set at the .05 level. Logistic regression was used to assess performance for individual motor features and their combination. We started by using the features that most strongly differentiated the two groups, then selected the feature leading to the best AUC performances. This commonly used type of greedy approach helped address the statistical challenges of high dimensional data. Leave-one-out cross-validation was used to evaluate the generalization performance of models, as recommended in the case of relatively small sample size^70^. Scikit-learn^71^ implementations LogisticRegression and GridSearchCV were used to define models and find optimal parameters for each set of motor features. Span of evaluated hyperparameters include: “C” in [0.01, 100], “penalty” in [l1, l2, none], “dual” in [True, False], “fit_intercept” in [True, False], and “solver” in [liblinear, lbfgs]. During the training process, we addressed class imbalance by up-sampling the minority group. Models used for prediction were evaluated using receiver operator curve characteristic (ROC) area under the curve (AUC) with 95% confidence intervals computed by the Hanley McNeil method^72^. Statistics were calculated in Python using SciPy low-level functions V.1.4.1, Statsmodels V.0.10.1, and Pingouin V.0.3.4^73–75^. Spearman’s rho correlation was used to assess the relationship between motor features and clinical variables, with statistical significance computed using a Student’s t-distribution.

### Reporting summary

Further information on research design is available in the Nature Portfolio Reporting Summary linked to this article.

## Supplementary information

Supplementary Material

Nature Research reporting summary

## Data Availability

Data that support the findings of this study are available from the corresponding authors upon request and following IRB rules and privacy regulations.

## Code availability

Custom code that supports the findings of this study is available from the corresponding authors upon request.
