# Supplementary material for: A tablet-based game for the assessment of visual motor skills in autistic children
Source: NPJ Digit Med. 2023 Feb 3;6:17. doi: 10.1038/s41746-023-00762-6 (PMC9898502; doi:10.1038/s41746-023-00762-6)
Supplement: Supplementary file 1 — Supplementary Material [file 41746_2023_762_MOESM1_ESM.pdf]

A TABLET-BASED GAME FOR THE ASSESSMENT OF VISUAL MOTOR SKILLS IN AUTISTIC CHILDREN: SUPPLEMENTARY MATERIAL

Sam Perochon[1,2], J. Matias Di Martino[1], Kimberly L.H. Carpenter[3,4], Scott Compton[3,4], Naomi Davis[3], Steven Espinosa[5], Lauren Franz [3,4,6], Amber D. Rieder[3,4], Connor Sullivan[3,4], Guillermo Sapiro[1, 7,+,*] and Geraldine Dawson[3,4 +,*]

[1]Department of Electrical and Computer Engineering, Duke University, Durham, NC, USA.

[2] Ecole Normale Supérieure Paris-Saclay, Gif-Sur-Yvette, France.

[3] Department of Psychiatry and Behavioral Sciences, Duke University, Durham, NC, USA.

[4] Duke Center for Autism and Brain Development, Duke University, Durham, NC, USA.

[5]Office of Information Technology, Duke University, Durham, NC, USA.

[6]Duke Global Health Institute, Duke University, Durham, NC, USA.

[7]Department of Biomedical Engineering, Mathematics, and Computer Sciences, Duke University, Durham, NC, USA.

[+]Equal senior authors

*Corresponding authors: Geraldine Dawson geraldine.dawson@duke.edu and Guillermo Sapiro guillermo.sapiro@duke.edu.

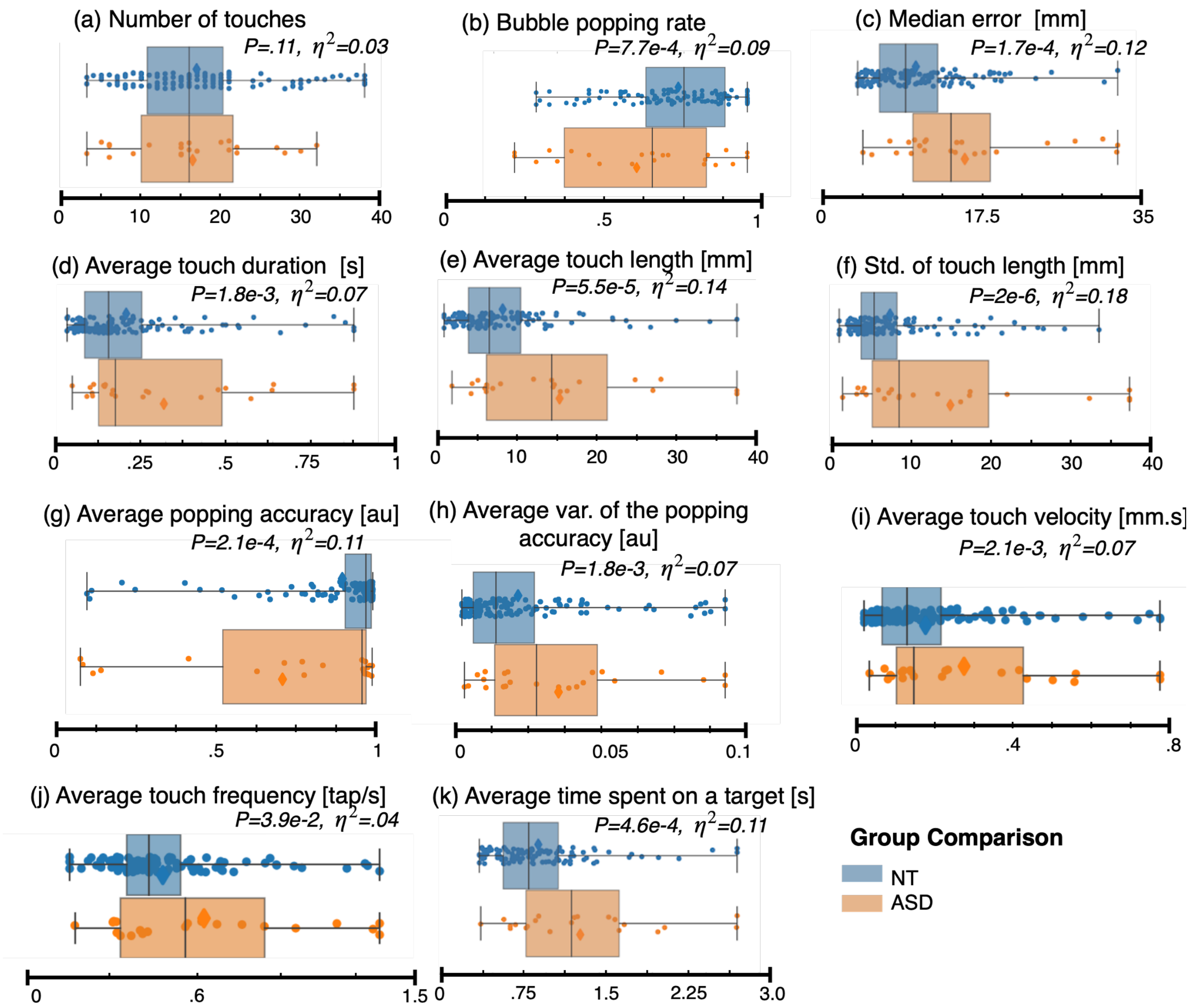

Supplementary Figure 1: Boxplot of the distribution of a few touch-related features for autistic versus neurotypical children, for the study 1 sample. Analysis was performed matching participants' age and experience across the neurotypical and autistic groups. These motor-related features show statistically significant differences between autistic and neurotypical toddlers (except for the *number of touches*). The extracted features presented here are detailed in the features extraction section. P-values were corrected using Benjamini-Hochberg procedure to control for FDR. Effect sizes are denoted as η^2. The line within the boxplot represents the median, the box represents the interquartile range, and the whiskers show extreme values. Scatter points show feature values for each participant.

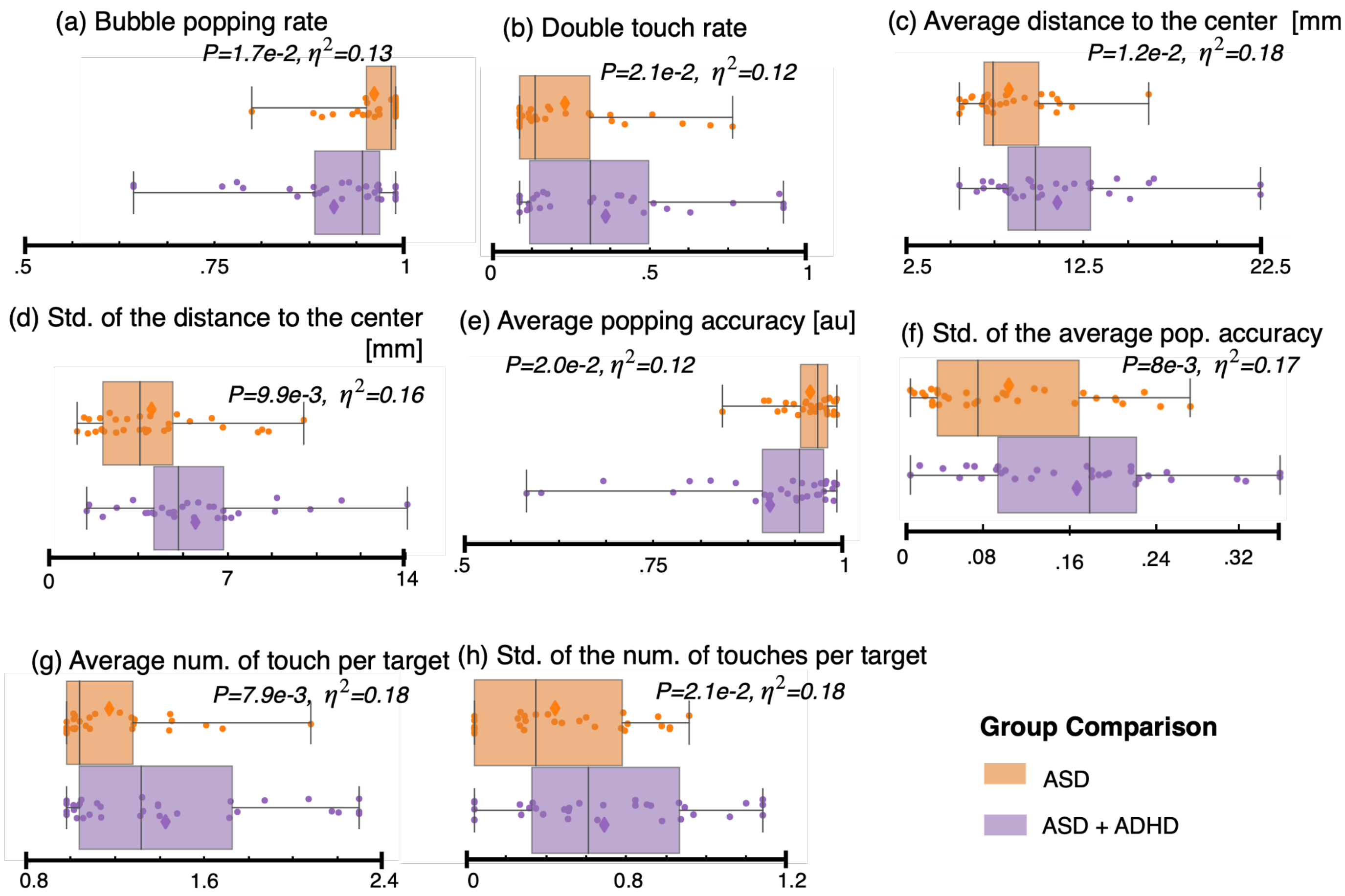

Supplementary Figure 2. Boxplot of the distribution of a few touch-related features for autistic children and those co-occurring ADHD, for the study 2 sample. Analysis was performed matching participants' age, IQ, and experience across the autistic participants with and without ADHD. These motor-related features show statistically significant differences between autistic participants with and without ADHD (except for the *number of touches*). The extracted features presented here are detailed in the features extraction section. P-values were corrected using Benjamini-Hochberg procedure to control for FDR. Effect sizes are denoted as η^2. The line within the boxplot represents the median, the box represents the interquartile range, and the whiskers show extreme values. Scatter points show feature values for each participant.

Applied force computation - Algorithms

Algorithm 1: Computation of a proxy for the force applied.

Input: =X(t), Y(t) and Z(t) time series of the acceleration of the iPad. Child's touchscreen information (Ti)i[1,N].

Output: Energy (Ei)i[1,N] associated with each child touch.

For each touch Ti of the child:

```
# Find beginning and ending timestamps of the dynamical response of the iPad
ti, tf = retrieve_touch_timestamps(Ti, X(t), Y(t), Z(t)) # See Alg. 2

# Compute the energy of the iPad associated to this touch
Ei =titf(X2+Y2+Z2)dt
```

Algorithm 2: retrieve_touch_timestamps

Input: = Ti single child's touch information, and X(t), Y(t), Z(t) the accelerations of the iPad.

Output: ti, tf beginning and ending timestamps of the dynamical response of the iPad.

```
# Initialize ti and tf to be the touch timestamps
ti = Tifirst timestamps
tf = Tilast timestamps

# Compute Z(t) standard deviation during the touch (orthogonal direction of the screen)
Z(t) = STD(Z(t), ti, tf)

# Looking for the final timestamps  tf  as the ending of the device's dynamical relaxation, by finding when Z(t) stays less than 0.5*Z(t)
tf = retrieve_final_timestamps(Z(t)[ti,tf], Z(t))
```

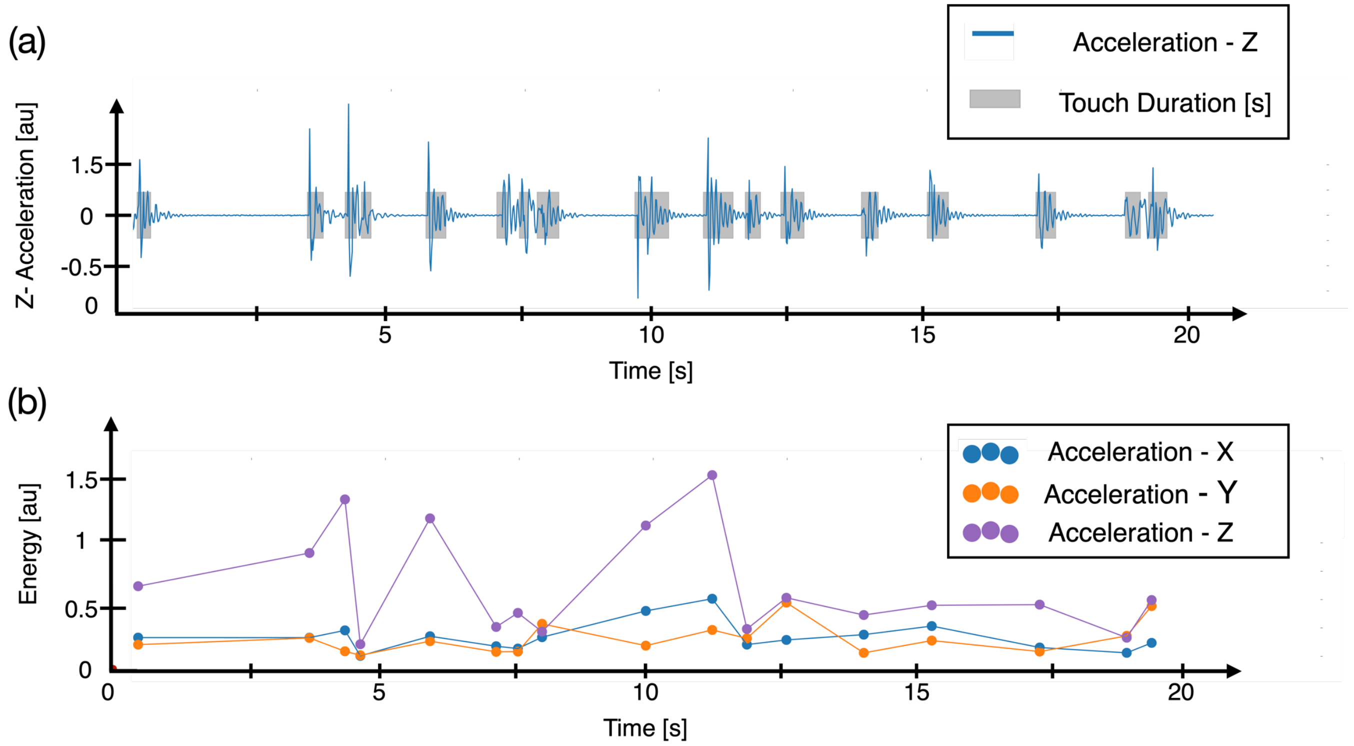

Supplementary Figure 3. (a) Example of the Z-acceleration (orthogonal direction of the screen) of the iPad during the game, with duration of the child's touches represented. (b) Example of the computed iPad energies. To compute a proxy for the force engaged by the child when touching the screen, we integrated the acceleration signal - indicative of the device's dynamical response to a touch - over the duration of a touch (grey shades), and then sum over the X, Y and Z components, as explained in the algorithms 1 and 2.

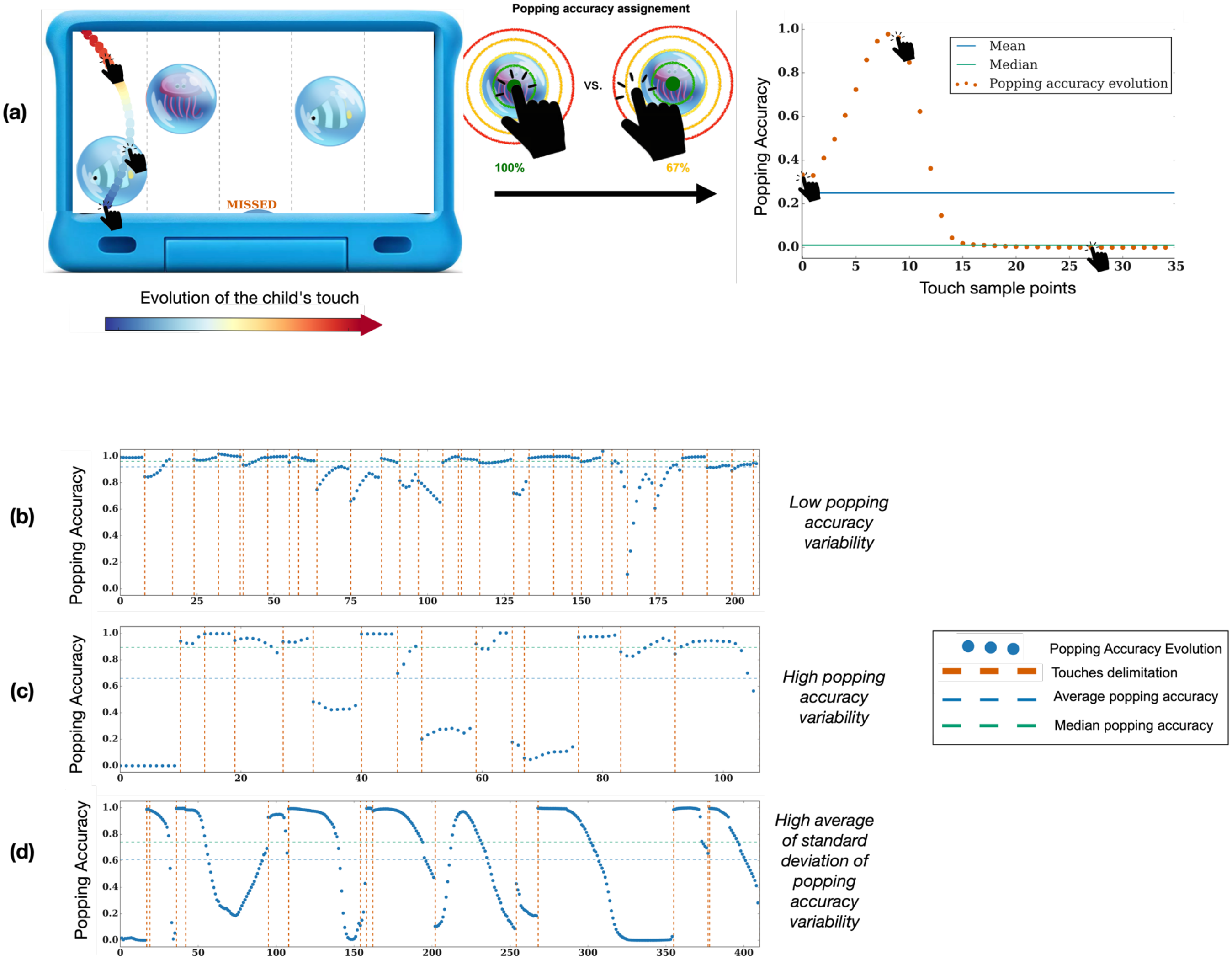

Supplementary Figure 4. a) Illustration of the *popping accuracy* assignment for all the sample points of a touch. (b-c-d) *Popping accuracy* evolution for three different participants. The *popping accuracy* provides information about the evolution of the accuracy of a child while their finger is touching the screen. a) Each sample point of a child's touch was assigned a score between 0 and 1 reflecting its closeness to the bubble. b) This participant showed high *popping accuracy* across their touches, low intra-touches variability (*average variation of the popping accuracy*), and low inter-touches variability (*variability of the average popping accuracy)*. c) This participant showed medium *popping accuracy*, low intra-touches variability, but high inter-touches variability. d) This participant showed medium *popping accuracy*, high intra-touches variability, and low inter-touches variability.

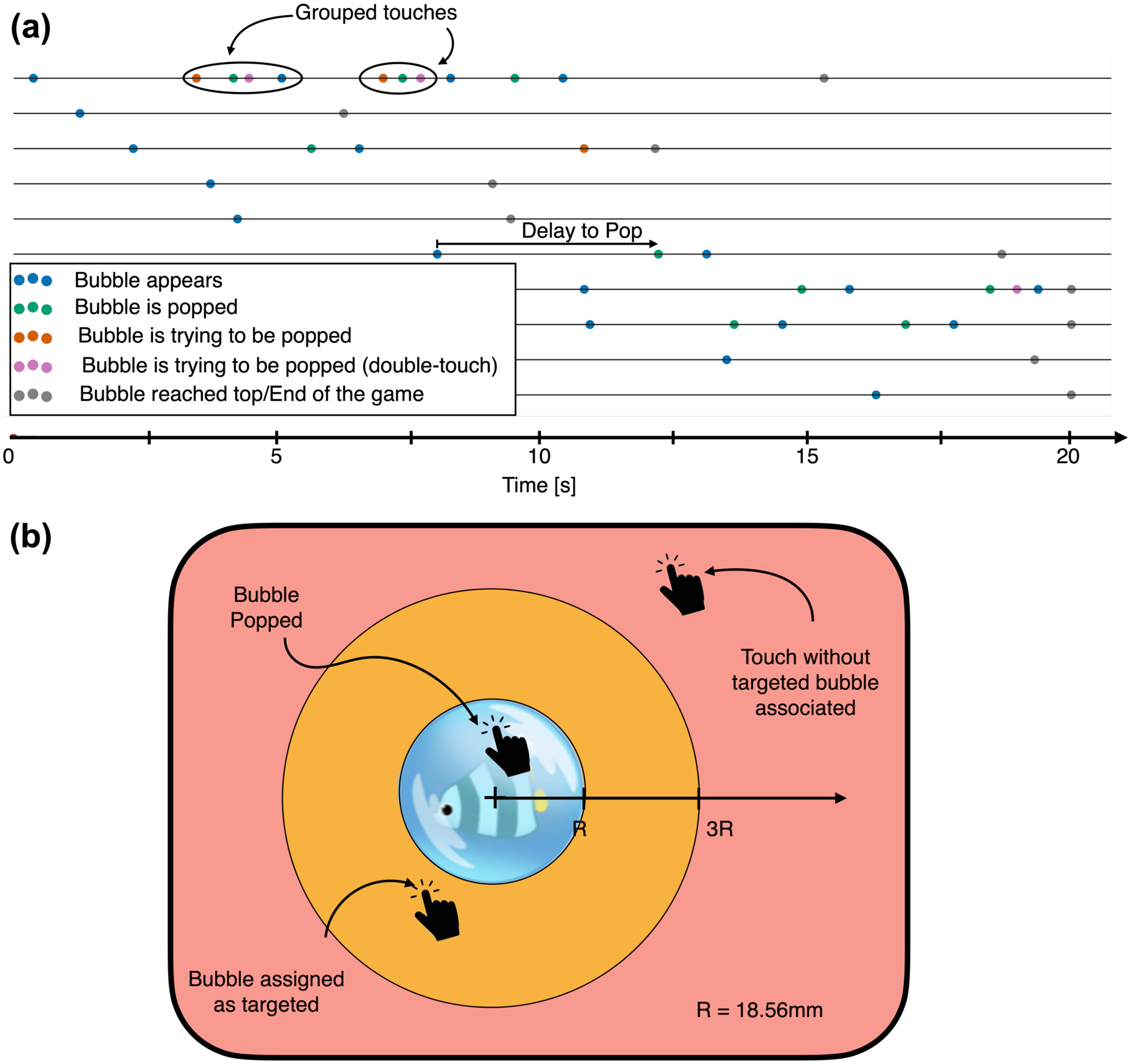

Supplementary Figure 5. (a) Example of a chronogram of gameplay events. (b) Diagram depicting how a touch was assigned to a bubble. (a) The grouped touches correspond to several touches intended to touch the same bubble. We assumed that a touch was intended to touch a specific bubble if the distance between the edge of that bubble and the touch onset location was less than 3.71cm, corresponding to 2R. Sub-figure (b) illustrates how we made the association between a touch and a bubble.

Supplementary Table 1: AUCs obtained by the model when using three motor features, by identified sex, race, and ethnicity

Subgroups	AUC [95% CI]	
	Study 1 (N=151)	***Study 2 (N=82)***
All	0.73 [0.63, 0.83]	0.74 [0.62, 0.86]
Sex		
Male	0.72 [0.60, 0.84]	0.78 [0.66, 0.92]
Female	00.76 [0.60, 0.92]	0.66 [0.41, 0.91]
Ethnicity		
Not Hispanic/Latino	0.75 [0.64, 0.86]	0.72 [0.59, 0.85]
Hispanic/Latino	0.71 [0.45, 0.97]	1.00 [1.0, 1.0]
Race		
Black or African American	0.71 [0.55, 0.87]	1.0 [1.0, 1.0]
White/Caucasian	0.77 [0.65, 0.89]	0.79 [0.65, 0.93]
All Other Races	0.72 [0.52, 0.92]	0.59 [0.24, 0.94]

The AUC values were relatively consistent across groups; however, confidence intervals were larger due to the smaller sample sizes. Leave-one out cross-validation approach was used. Features used to fit the model was the average *length,* the *average touch duration*, and the a*verage time spent* for the Study 1 sample, and the *average distance to the center , the number of targets*, and the *screen exploratory percentage* for the study 2 sample.
